# In Vitro and in Vivo Study of Poly(Lactic–*co*–Glycolic) (PLGA) Membranes Treated with Oxygen Plasma and Coated with Nanostructured Hydroxyapatite Ultrathin Films for Guided Bone Regeneration Processes

**DOI:** 10.3390/polym9090410

**Published:** 2017-09-02

**Authors:** Daniel Torres-Lagares, Lizett Castellanos-Cosano, María Ángeles Serrera-Figallo, Francisco J. García-García, Carmen López-Santos, Angel Barranco, Agustín Rodríguez-Gonzalez Elipe, Cristóbal Rivera-Jiménez, José-Luis Gutiérrez-Pérez

**Affiliations:** 1 Faculty of Dentistry, University of Seville. Avicena Street, 41009 Seville, Spain; lizettcastellanos@yahoo.es (L.C.-C.); cmriveraj@gmail.com (C.R.-J.); jlgp@us.es (J-L.G.-P.); 2 Institute of Materials Science of Seville (CSIC-University of Seville), Américo Vespucio Street n 49, 41092 Seville, Spain; franciscoj.garcia@icmse.csic.es (F.J.G.-G.); mclopez@icmse.csic.es (C.L.-S.); angel.barranco@csic.es (A.B.); arge@icmse.csic.es (A.R.-G.E.)

**Keywords:** guided bone regeneration, hydroxyapatite, PLGA, oxygen plasma treatment, magnetron sputtering

## Abstract

The novelty of this study is the addition of an ultrathin layer of nanostructured hydroxyapatite (HA) on oxygen plasma modified poly(lactic–*co*–glycolic) (PLGA) membranes (PO_2_) in order to evaluate the efficiency of this novel material in bone regeneration. Methods: Two groups of regenerative membranes were prepared: PLGA (control) and PLGA/PO_2_/HA (experimental). These membranes were subjected to cell cultures and then used to cover bone defects prepared on the skulls of eight experimental rabbits. Results: Cell morphology and adhesion of the osteoblasts to the membranes showed that the osteoblasts bound to PLGA were smaller and with a lower number of adhered cells than the osteoblasts bound to the PLGA/PO_2_/HA membrane (*p* < 0.05). The PLGA/PO_2_/HA membrane had a higher percentage of viable cells bound than the control membrane (*p* < 0.05). Both micro-CT and histological evaluation confirmed that PLGA/PO_2_/HA membranes enhance bone regeneration. A statistically significant difference in the percentage of osteoid area in relation to the total area between both groups was found. Conclusions: The incorporation of nanometric layers of nanostructured HA into PLGA membranes modified with PO_2_ might be considered for the regeneration of bone defects. PLGA/PO_2_/HA membranes promote higher osteosynthetic activity, new bone formation, and mineralisation than the PLGA control group.

## 1. Introduction

The use of physical barriers to prevent the invasion of gingival and connective tissue cells into bone cavities during the healing process is called guided bone regeneration (GBR) [1]. Tissue engineering enables the fabrication of membranes that mimic the structural properties of the original tissues, providing stable support to the extracellular matrix [2]. The application of polymeric biodegradable materials has become a common practice, however there is limited knowledge of the behaviour of the osteoblastic cells in them [3,4]. Poly(lactic–*co*–glycolic) (PLGA) copolymers, of all of the existing membranes, show good bone adhesion, vascularity, biodegradability and the ability of osteoblastic cells to grow on the surface of in vitro cultivations [5]. PLGA is known to be degraded by hydrolysis and eliminated in the Krebs cycle in the form of carbon dioxide and water. Therefore, it is successfully used as a biodegradable membrane in GBR. Its hydrophobicity and relative low cellular affinity, however, limit its choice [6].

As is well known, internal cellular organisation and orientation are controlled by focal adhesions. These focal adhesions mediate the regulatory effects of adhesion of the extracellular matrix and the distribution of actin-myosin fibres depending on surface properties [7]. Also, a substantial increase in osteoblastic mitochondrial bioenergy is oriented to focal adhesions, which are formed during cell adhesion and are constantly inserted and uninserted with the movement of the cell. Therefore, these focal adhesions based on integrins serve as mechanosensors, converting the mechanical signals of the medium into biological signals [8,9]. This scientific and biological evidence has allowed us to consider that the induction of osteoblast cellular activity can be achieved by modifying the surface of a biomaterial.

One of the most effective processes for the modification of PLGA polymer surfaces is the oxygen plasma surface modification [10]. The oxygen plasma treatment increases the polymer roughness and introduces surface chemical functionalizations, stimulating the adhesion of osteogenic mediators and cells and speeding up the membrane degradation [11]. Therefore, we may imply that the osteoinductive capacity of the barrier could be optimised by adding fine layers of nanocomposite particles, which might promote the osteoblastic adhesion and induce a functional differentiation that enables the formation of new bone [12,13].

A nanocomposite particle such as hydroxyapatite (HA) (of synthetic origin) may act as a membrane surface modifier. HA has a composition and mechanical resistance similar to natural bone, as well as osteoconductivity, osteoinductivity and biodegradability. Furthermore, medical products such as screws, plates and cylinders made of this material have been shown to form a natural bond with the bone in vivo [14]. Therefore, including HA within a biomaterial may allow us to regulate its pH, preventing inflammatory reactions and inducing bone formation [15].

The novelty of this study is the addition of a thin nanostructured layer of HA by magnetron sputtering on oxygen plasma modified PLGA foils (PO_2_) to evaluate the efficiency of the resulting membranes in bone regeneration. The initial hypothesis was that modified PLGA membranes were able to alter the potential for bone regeneration when compared to PLGA.

## 2. Materials and Methods

### 2.1. Preparation of the Membranes

Thirty 50 μm thick resorbable inert PLGA scaffolds based on poly(lactic–*co*–glycolic) acid were fabricated using polycondensation (Institute of Materials Science, Seville, Spain). The membranes were used to cover bone defects prepared on the skulls of eight experimental rabbits. After subduing the membranes to cell culture to identify their viability, two groups of regenerative membranes (*n* = 4 each) were prepared and tested for GBR processes: (1) PLGA (control) and (2) PLGA/PO_2_/HA (experimental).

**Figure 1:**
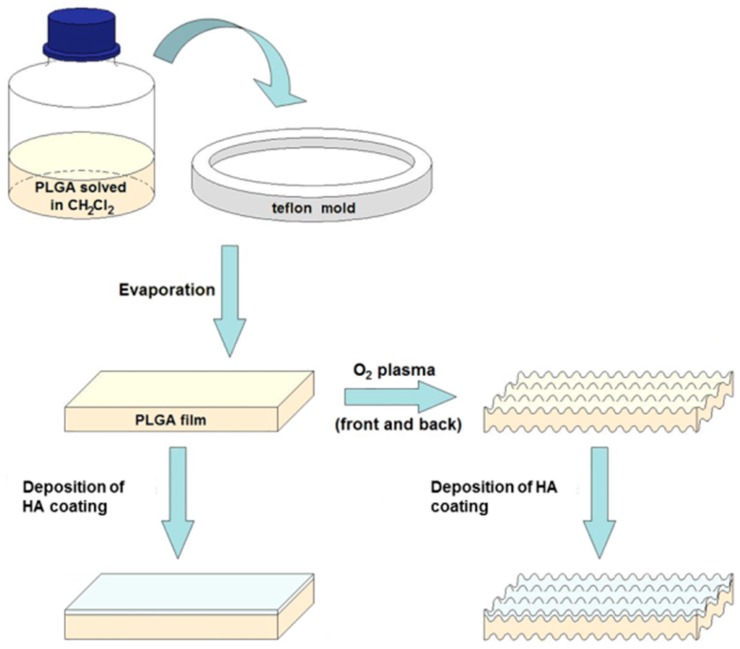
Manufacturing process of Poly(lactic–*co*–glycolic) PLGA membranes and subsequent surface treatment and deposition of thin layers of hydroxyapatite.

For the preparation of the control membrane, 10 mL of a solution of PLGA (PLGA pellets with a copolymer ratio of 75:25 (lactic/glycolic acid) from Sigma-Aldrich Inc., St. Louis, MO, USA) in 1.5% dichloromethane was prepared by evaporation of the solvent on a Teflon plate for 48 h in air at room temperature obtaining a film of suitable consistency and a thickness of about 50 μm [16]. Figure 1 shows a membrane preparation schematic, as well as the possible surface treatment with oxygen plasma and the surface coating with hydroxyapatite.

The control group of PLGA membranes was characterised by X-ray photoemission spectroscopy (XPS, ESCALAB 250Xi, ThermoFisher Scientific, Waltham, MA, USA) so that the following chemical composition was registered on the membranes’ surfaces—54% carbon and 46% oxygen. The molecular weight of the PLGA copolymer was 12 kDa. The PLGA was synthesised by means of the ring-opening copolymerisation of two different monomers, the cyclic dimers (1,4-dioxane-2,5-diones) of glycolic acid and lactic acid. During polymerisation, successive monomeric units (of glycolic or lactic acid) were attached to PLGA using ester linkages, thereby yielding linear, aliphatic polyester.

Subsequently, the membranes of Group 2 were coated with bioactive layers of HA by excited magnetron sputtering using a radiofrequency power of 50 W under an argon atmosphere at a pressure of 3 × 10^−3^ mbar [17]. Before deposition, the chamber was maintained at a base pressure of 1 × 10^−6^ mbar. The sputtering deposition was carried out using a copper coating that was 3 mm thick and 46 mm diameter white calcium phosphate. (HA) target (hydroxyapatite) was 4 mm thickness, 46 mm in diameter and 99.9% purity (Kurt J. Lesker Company, Jefferson Hills, PA, USA). The target sample distance was 10 cm and the deposition time was 40 min, obtaining a thickness of the coating of HA on PLGA of around 15 nm.

### 2.2. Materials Characterizations

The surface chemical composition of the samples was analysed by X-ray photoelectron spectroscopy (XPS) using an ESCALAB 210 spectrometer (Thermo Fisher Scientific, Waltham, MA, USA), operating at constant pass energy of 20 eV. Nonmonochromatized Mg Kα radiation was used as the excitation source. The atomic surface concentrations were quantitatively determined from the area of C1s, O1s, P2p and Ca2p peaks. A Shirley-type background was subtracted, and the peak areas were corrected by the electron escape depth, the spectrometer transmission and the photoelectron cross-sections.

Fourier transform infrared (FTIR) spectra were collected in a JASCO FT/IR-6200 IRT-5000 (Oklahoma, OK, USA) under vacuum conditions and specular reflectance mode.

The surface topography of the films was characterised by noncontact atomic force microscopy (AFM) with a Cervantes AFM system from NANOTEC (Feldkirchen, Germany) using commercial noncontact AFM tips from MikroMasch (Wetzlar, Germany). The surface of the membranes was also studied by scanning electron microscopy (SEM) in a Hitachi S4800 field emission microscope (Tokyo, Japan) of the coating of HA on PLGA of around 15 nm.

### 2.3. In Vitro Cell Cultures

The assays were performed using the human osteoblast line MG-63 acquired from the Center for Scientific Instrumentation (CIC) at the University of Granada (Granada, Spain). The MG-63 line shows faster growth than the primary bone-forming lines but retains quite a few characteristics of these, which makes it a good model in vitro.

Firstly, a control of mycoplasma contamination was performed by PCR to verify that the cells were free of contamination. A method of detection by PCR (polymerase chain reaction) was used. The amplification of a band of approximately 500 bp was performed according to the species—specific to eight species of mycoplasma (*M. hyorhinis, M. arginini, M. pneumoniae, M. fermentans, M. orale, M. pirum, Acholeplasma laidlawii* and *Spiroplasma mirum*)—using a single pair of oligonucleotides corresponding to the 16S RNA. PCR was performed by taking an aliquot of the conditioned medium from the cells in culture after at least 48 h of culture [18,19]. MG-63 cells were cultured on control and experimental membranes.

For the determination of cell adhesion and osteoblast viability, osteoblasts were cultured on the PLGA/PO_2_/HA and the PLGA control membrane, in triplicate, planting 120,000 cells. At 24 h, the cultures were analysed by microphotography of osteoblasts [20]. Previously, the cells had been fixed with 70% ethanol for 5 min, as it was not possible to observe them without prior fixation. Bright-field microphotographs were taken of each condition at 5×, 10× and 20× with the Axio Observer A1 inverted microscope (Carl Zeiss). These images were also used to determine the average size of viable cells.

For the determination of the mitochondrial energy balance, the MitoProbe™ JC-1 Assay Kit was employed. JC-1 is a membrane permeable dye widely used for determining mitochondrial membrane potential in flow cytometry and fluorescent microscopy [21]. When mitochondria show good functioning, the probe accumulates in the mitochondria and forms aggregates, which emit in red (~590 nm). When the mitochondrial membrane potential decreases during cellular damage phenomena, the emission of the fluorescence turns green (~529 nm), decreasing the red/green ratio, because of the passage to the monomeric form of the probe. Osteoblast cultures were performed on the PLGA/PO_2_/HA and PLGA control membrane in triplicate, planting 120,000 cells. The cultures were analysed at 24 h. The observed red and green fluorescence was due to the JC-1 probe, after a 30 min incubation of the cells in culture. Images were taken with the 40× and 20× lenses of the Axio Observer A1 (Carl Zeiss, Oberkochen, Germany) fluorescence microscope.

For the determination of osteogenesis and the morphology of adherent osteoblasts, one staining was employed. This staining consisted of the use of phalloidin-TRITC—a fluorescent phallotoxin that can be used to identify filamentous actin (F-actin) [22]—along with the use of DAPI (4′,6-diamidino-2-phenylindole)—a nuclear and chromosome counterstain emitting blue fluorescence upon binding to AT regions of DNA cells attached to the different membranes for 24 h [23]. Osteoblast cultures were performed on the PLGA/PO_2_/HA and PLGA control membrane in triplicate, planting 120,000 cells. The cultures were analysed at 24 h. Cells were fixed with 70% alcohol. Both techniques were visualised at 20× with the fluorescence microscope Axio Observer A1 (Carl Zeiss).

### 2.4. Animal Experimentation Specimens

Four white, New Zealand-breed experimentation rabbits with identical characteristics (age: 6 months; weight: 3.5–4 kg) were selected for the study and fed daily with rabbit-maintenance Harlan-Teckland Lab Animal Diets (2030).

The surgical interventions were carried out at the Minimally Invasive Surgery Centre Jesús Usón (CCMI, Cáceres, Spain). The experiment was developed in accordance with the guidelines of the US National Institute of Health (NIH) and European Directive 86/609/EEC regarding the care and use of animals for experimentation. The study also complied with the European Directive 2010/63/EU about the protection of animals used for scientific purposes and with all local laws and regulations. The researchers obtained the approval of the Ethics Committee of the Minimally Invasive Surgery Centre Jesús Usón (CCMI, Cáceres, Spain). As required by the legislative framework, the minimum number of animals was used for ethical reasons [24]. Comparable models have been published concerning the histological and animal experimentation methods [6].

### 2.5. Surgical Procedure

**Figure 2:**
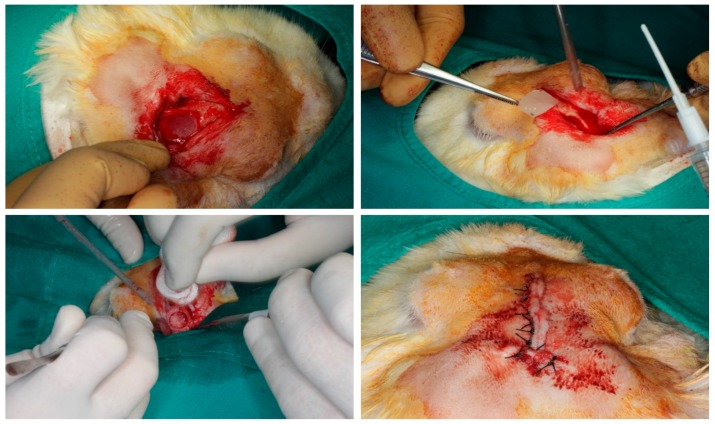
Surgical procedures in model animal.

The surgical procedure followed the same methodology as previously described [1,6]. The animals were immobilised, and their vital signs were checked. The anaesthesia used for induction was intravenous midazolam (0.25 mg/kg) and propofol (5 mg/kg). For maintenance, the animals inhaled 2.8% inspired sevoflurane gas. Analgesia was provided with ketorolac (1.5 mg/kg) and tramadol (3 mg/kg). After the rabbits were sedated and prepared, incisions between the bases of their ears and between their eyes were made with a No. 15 scalpel blade. After the two incisions were connected with an incision that coincided with the skull midline, a triangular field was discovered. The epithelial, connective, and muscular tissues were displaced using a Prichard periosteotome. The skull surface was washed with a sterile saline solution, maintaining aspiration. Two bone defects (diameter: 10 mm; depth: 3 mm) were created on the parietal bone, on each side of the skull midline, 3 mm apart, using a trephine (Helmut-Zepf Medical Gmbh, Seitingen, Germany) mounted on an implant micromotor operating at 2000 rpm under saline irrigation. The trephine had an internal diameter of 10 mm, a length of 30 mm, and teeth of 2.35 mm. Because the defects were created in a symmetric fashion in relation to the longitudinal axis of each rabbit skull, possible between-group variations in section thickness were minimised. For each defect, the outer table and the medullary bone were completely removed with piezosurgery, and the inner table was preserved to avoid damage to the brain tissue. The depth was controlled with a periodontal probe. A randomly assigned membrane was used to cover each bone defect. The randomisation sequence was generated using specific software (Research Randomizer, V. 4.0, Urbaniak GC & Plous S, 2013) [2]. The PLGA fibres of the barriers were also randomly oriented. The membranes were fixed with the fibrin tissue adhesive Tissucol (Baxter, Hyland S.A. Immuno, Rochester, MI, USA), which was placed on the bone rims adjacent to the defects. Proper adhesion and limited mobility of the membranes were confirmed when the flaps were moved back to their initial positions. Sutures were made on the following planes using resorbable material: periosteal (4/0), sub-epidermal (4/0) and skin (2/0). Simple stitches were used as close as possible to the edge. The wound was carefully cleaned with a sterile saline solution. Anti-inflammatory analgesia (buprenorphine 0.05 mg/kg and carprofen 1 mL/12.5 kg) was administered. The animals were sacrificed two months after surgery using an intravenous overdose of potassium chloride solution. Samples were obtained from the skull of each specimen, cutting them in an anatomical sagittal plane. After the brain mass was separated and the skull was washed with a sterile saline solution, the tissue samples were cut and marked individually. The complete surgical sequence is shown in Figure 2.

### 2.6. Comparison of Bone Density (BoneJ)

The mean bone density of the surgical bone defect created and then covered with a random membrane was assessed by micro computed tomography (micro-CT) [25].

**Figure 3:**
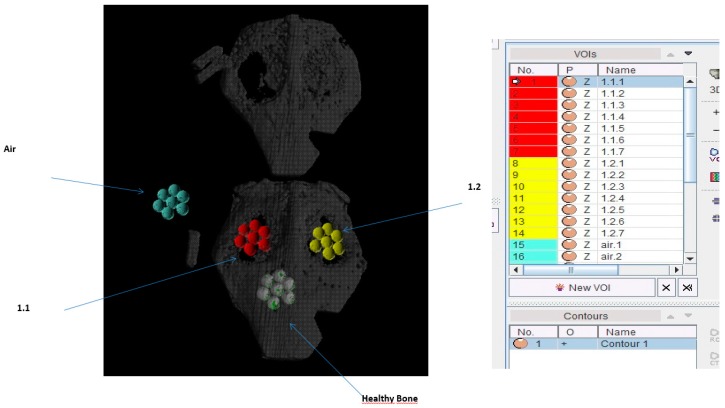
Microtomography study on animal skull.

To determine variations in bone density measurements due to manual positioning of sampling cylinders, three cylinders of identical shape were positioned independently according to a central localisation criterion within each lesion and to maximise the overlap with bony structures. Spheres of 2 mm in diameter were placed in a rosette arrangement with a manually adjusted arrangement to match the largest possible bone structure (Figure 3).

The application of a binary threshold (intensity of pixels) enables us to define structures of interest. For the initial test, a threshold value of 6500 was chosen for all samples. This value is probably quite high and may lead to the detection of more individual trabecular structures while ignoring structures with less density. Changes in this threshold can have significant effects on the results obtained. Bone image analysis in ImageJ can therefore be used to evaluate different properties of the bone through a series of BoneJ plugins. The BoneJ plugin provides free, open-source tools for trabecular geometry and whole bone shape analysis [26].

### 2.7. Histological Processing of Samples

The processing of samples followed the protocol previously described [27]. Cranial blocks from containing fixtures were retrieved and stored in a 5% formaldehyde solution (pH 7). The blocks were retrieved from the regenerated bone defect using an oscillating autopsy saw (Exakt, Kulzer, Wehrheim, Germany). The dissected specimens were immediately immersed in a solution of 4% formaldehyde and 1% calcium and processed for ground sectioning following the Donath and Breuner method [28].

For histological staining and rapid contrast tissue analysis (Merck Toluidine Blue-Merck, Darmstadt, Germany), a metachromatic dye was used to assess the percentage of new bone formation. A 1% toluidine blue (TB) solution with a pH of 3.6 was chosen and adjusted with HCl 1 N. The samples were exposed to the dye for 10 minutes at RT, rinsed with distilled water, and air-dried. The von Kossa (VK) silver nitrate technique (Sigma–Aldrich Chemical Co., Poole, UK) was applied to visualise the mineralised bone [6].

### 2.8. Statistical Analysis

Means and standard deviations (SD) were calculated. The intraexaminer reliability was assessed using the Kappa test. Since the Kolmogorov–Smirnov test demonstrated that the data were not normally distributed, the Kruskal–Wallis test was run for post hoc comparisons. The level of significance was set in advance at *p* ≤ 0.05 [6]. The Statview F 4.5 Macintosh software (Abacus Concepts, Berkeley, CA, USA) was used for the analysis [2]. All the statistical probes applied in this study adhere to the requirements for oral and dental research [29].

## 3. Results

The C1s spectrum of the bare PLGA consisted of three well-defined bands at 284.6, 287.5 and 288.5 eV—these bands are attributed to C–C/C–H, C–O and C=O/COOH functional groups in the polymer [30,31]. In addition, these membranes have a hydrophobic character demonstrated by a contact angle with water of 99° [32]. The morphology of PLGA membranes is quite flat, with an RMS roughness value of less than 1 nm obtained by analysis of surface topography through atomic force microscopy (AFM).

**Figure 4:**
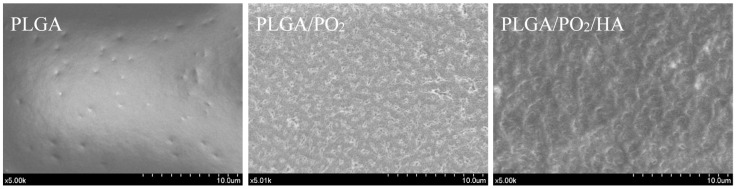
Scanning Electron Microscopy (SEM) images of the three steps of the membrane modification scheme.

The membrane surfaces in Group 2 were first exposed to pure oxygen plasma for periods of 30 min in a parallel plate capacitive RF reactor working at a pressure of 0.1 mbar. An RF power of 10 W was applied to the top electrode and a self-induced negative bias voltage of 200 V was generated on the bottom electrode, acting as sample holder. This treatment modifies the surface tension state, rendering the PLGA membrane hydrophilic [33]. The treatment of PLGA substrates with oxygen plasma took place at close to ambient temperatures (RT: 23.0 ± 1.0 °C) and did not affect their structural integrity [33]. This process generates an engraving effect, improving the roughness of the surface and favouring the adhesion of oxide layers to PLGA substrates. Figure 4 shows the macroscopic appearance of the PLGA membranes before and after surface treatment with oxygen plasma. Additional information about the PLGA membranes after and before the oxygen plasma treatments can be found elsewhere [1,2,30,31,32,33].

Surface topography analysis also enables us to appreciate the considerable increase in surface roughness of the PLGA membrane after exposure to oxygen plasma, with an RMS roughness value greater than 300 nm, without affecting structural integrity. This was an intermediate step to enhance the adhesion of the HA nanostructured film [31].

**Figure 5:**
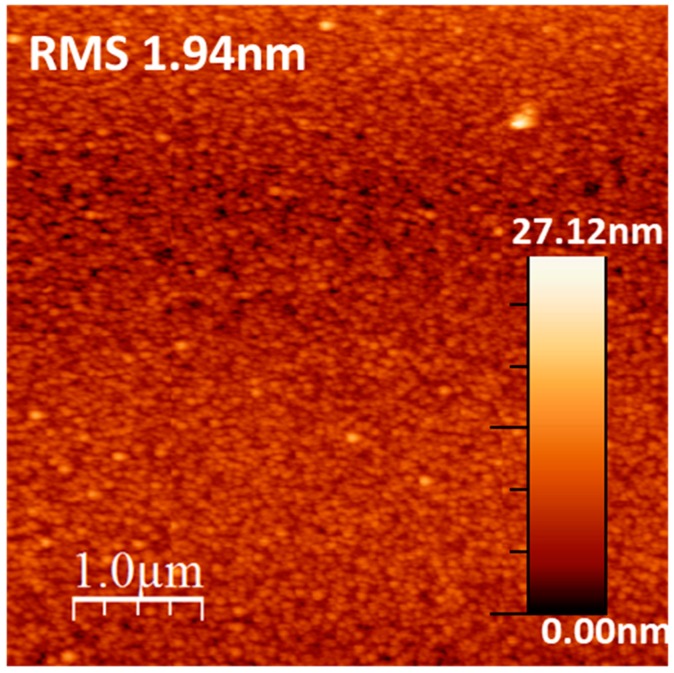
1 μm × 1 μm AFM micrograph of the hydroxyapatite (HA) surface with a RMS roughness coefficient of 1.94 nm.

**Figure 6:**
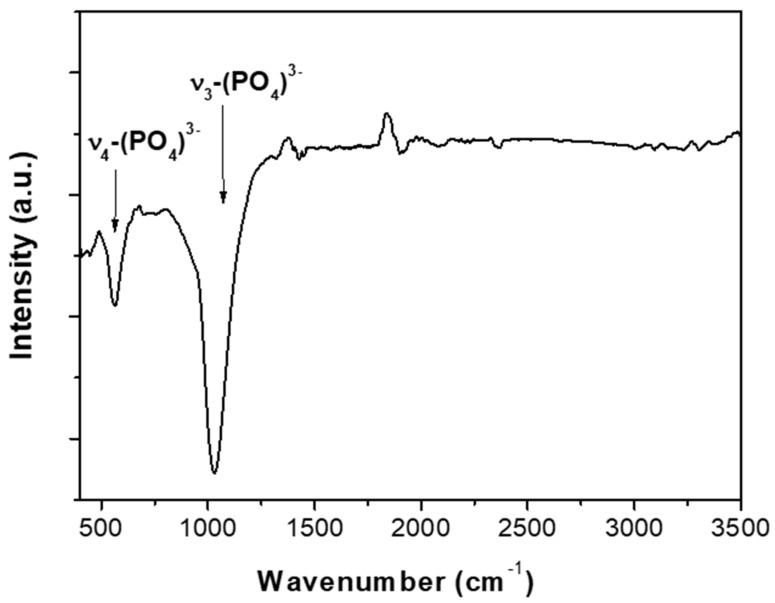
Fourier transform infrared (FTIR) spectrum of the HA coating.

**Figure 7:**
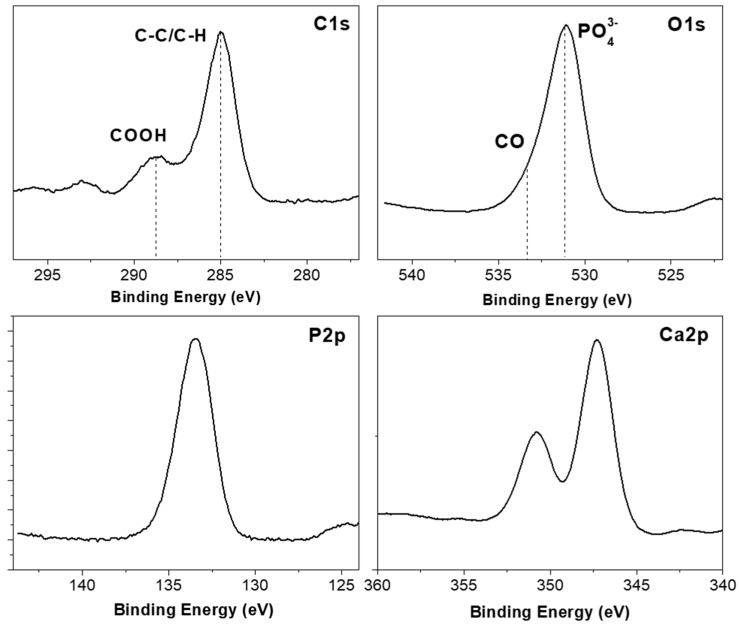
High resolution XPS spectra of the HA surface: carbon C1s, oxygen O1s, phosphorus P2p and calcium Ca2p regions. Guidelines mark the main functional groups.

A thickness of the coating of HA on PLGA around 15 nm and a RMS roughness coefficient around 2 nm was obtained, as shown in Figure 5. Additionally, the AFM surface topography image of the HA coating shows a smooth and uniform microstructure composed of grain-like features (Figure 5). Fourier transform infrared spectroscopy (FTIR) has confirmed the presence of phosphate functional groups (Figure 6). In particular, the broad absorbance band at 1031–1042 cm^−1^ corresponding to the (υ_3_) asymmetric vibration lines of (PO_4_)^3−^ group indicates an amorphous Ca–P structure [34]. Moreover, the study of the HA surface chemical composition by means of X-ray photoelectron spectroscopy (Figure 7) enables identification of the typical chemical states of the components in an ideal hydroxyapatite surface. Also, the estimated Ca/P ratio of 1.34 (see Table 1 with the atomic composition percentages) is in good agreement with other quasi-stoichiometric HA structures obtained by magnetron sputtering techniques in an inert atmosphere [35].

**Figure 8:**
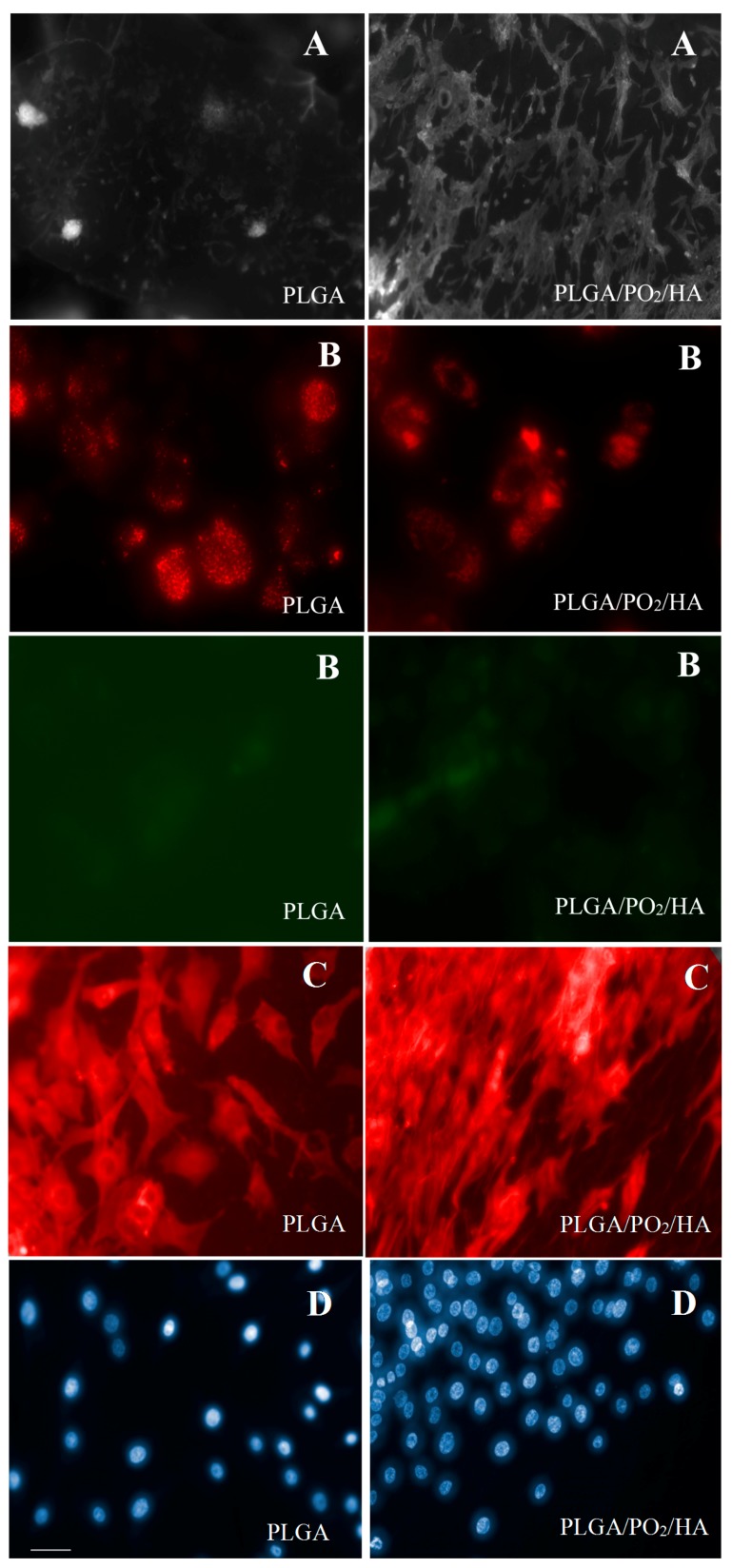
(**A**) Microphotography of osteoblasts on the membranes of PLGA and PLGA/PO_2_/HA; (**B**) JC-1 Probe Marking Microphotography; (**C**) Staining with phalloidina-TRITC 50 μg/mL; (**D**) Staining with DAPI. Bar in D-PLGA = 10 microns (same scale for all images). Images taken with the fluorescence microscope Axio Observer A1 (Carl Zeiss).

The main study findings on cell cultures are shown in Table 2 and Figure 8. The results obtained in relation to cell morphology and adhesion of osteoblasts to the membranes show that the osteoblasts bound to PLGA were smaller in size and with a lower number of adhered cells, with fewer extensions and filopodia than the osteoblasts bound to the PLGA/PO_2_/HA membrane (*p* < 0.05). Also, it was observed that the PLGA/PO_2_/HA membrane had a higher percentage of viable cells bound than did the PLGA control membrane (*p* < 0.05).

Quantification by flow cytometry of the JC-1 marker was used as a measure of mitochondrial potential. The red/green ratio for the osteoblasts that adhered to the PLGA/PO_2_/HA membrane (2.57) was higher when compared to the PLGA control membrane (1.69), which would indicate a better energy balance of the cells that adhered to the PLGA/PO_2_/HA membrane, but no statistical significance was observed (*p* > 0.05) (Figure 8).

When determining the degree of osteogenesis and morphology of osteoblasts adhered by F-actin staining and bright-field images, it was observed that the PLGA/PO_2_/HA membrane showed cells with a larger area, although no statistically significant differences were obtained (*p* > 0.05).

When comparing the obtained data from the BoneJ trabecular analysis plugins, statistically significant differences were observed for numerous variables between the PLGA control membrane and the PLGA/PO_2_/HA experimental membrane (*p* < 0.05). The data obtained from the bone density analysis are summarised in Table 3.

**Figure 9:**
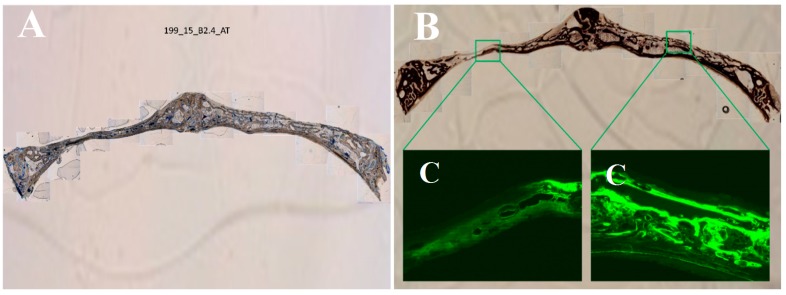
Histology images. (**A**) Toluidine blue; (**B**) Von Kossa and (**C**) Fluorescence. Distance from one side of the bone fragment to the other ≈35 mm. Left side is control (PLGA).

The histology analysis (Figure 9) only showed a statistically significant difference for the percentage of osteoid area in relation to the total area between the PLGA control membrane and the PLGA/PO_2_/HA membrane (Table 4).

## 4. Discussion

Cells that depend on their adhesions and the interactions they have with the underlying substrate structures and extracellular matrix maintain their functionality [36]. The underlying microenvironment provides a means by which the cells move orient and differentiate to form different types of cells and therefore tissues [37]. The structural and topographic characteristics of PLGA provide improved adhesive potential and ability to migrate and bond at the cell-substrate interphase on osteoblasts [1].

Previous studies have shown that modifying PLGA membranes with oxygen plasma may improve the degradation degree of the PLGA scaffold [2,6,10,38].

Other studies have found that HA increases the biodegradation rate of the membranes due to higher hydrophilicity and neutralisation against acidic degradation products of PLGA, and it has also shown excellent capabilities of calcium collection and bone-like apatite formation with great osteoconductivity [14,39,40]. Therefore, both modifier mechanisms might improve the degradation of the barriers enhancing bone healing. In our study, the functionalised membranes with PO_2_ and HA particles show a significantly superior efficacy for bone regeneration to the untreated barriers, confirming the initial hypothesis.

Over the past few decades, calcium phosphate (Ca_3_(PO_4_)_2_) ceramics have been widely used as bone graft substitutes, mostly due to the similarity of their chemical composition with the mineral phase of bone. Many types of calcium phosphates have been considered as biomaterials for bone reconstruction in dentistry, orthopaedics and maxillofacial surgery due to the different behaviours that each one exhibits in the living organism, including bioactivity, biodegradability and biological response [41]. The bioactivity, degradation behaviour and osteoconductivity/osteoinductivity of calcium phosphate ceramics generally depend on the calcium/phosphate ratio, crystallinity and phase composition [42]. The synthetic HA (Ca_10_(PO_4_)_6_(OH)_2_) shows good stability in the body, whereas tricalcium phosphates (α-TCP, β-TCP, Ca_3_(PO_4_)_2_) are more soluble. BCP (a mixture of HA and β-TCP) has intermediate properties depending on the weight ratio of stable/degradable phases. Therefore, the dissolution rate decreases in the following order: α-TCP > β-TCP > BCP > HA [42]. Due to their nature, Ca_3_(PO_4_)_2_ ceramics also exhibit high biocompatibility and ability to bind with bone tissue under certain conditions—however, given their fragility, their clinical applications have been limited to non-carrier or low-load parts of the skeleton [43]. In fact, it is thought that nanoparticles of hydroxyapatite (nHA) are one of the most promising bone graft materials due to their ability to mimic the structure and composition of natural bone [44]. The HA used in this study is a synthetic material of the same composition as the HA present in the human organism Ca_5_(PO_4_)_3_ (OH). Synthetic HA as a material for GBR has a long history of use, and its results are excellent [45]. Almost all materials or scaffolds used in GBR base their composition on HA, the main component of mineralised connective tissue [46].

In vitro cell cultures in our study showed osteoblasts with a larger size, a higher number of adhered cells with more extensions and filopodia, and a higher percentage of viable cells bound to the PLGA/PO_2_/HA membrane (*p* < 0.05). These results are consistent with those of previous published studies, obtaining an improved in vitro mineralisation and in vivo osteogenesis capacity of composite scaffolds with an increase in both the viability and proliferation rate of cells [5,14].

In this study, all surgical procedures of bone extraction and membrane placement were performed by two surgeons. During the procedure, the membranes’ stabilities were guaranteed and did not show any displacement after implantation. The implanted biomaterial was well tolerated by the surrounding soft tissues and no evidence of necrosis, allergic reactions, immune reactions or incompatibility was observed after two months.

Both micro-CT and histological evaluations confirmed that the PLGA/PO_2_/HA membranes enhance bone regeneration. Our results agree with those of other previously published studies, where they even observed the formation of new cortex and recanalisation of the marrow cavity via inspection [15].

## 5. Conclusions

Within the limitations of this study, the following conclusions may be drawn:

(1) We have verified the incorporation of nanometric layers of nanostructured HA films into PLGA membranes modified with PO_2_ are effective for the regeneration of bone defects when applied to skull defects in an animal model. We have verified the incorporation of nanometric layers of nanostructured HA films into PLGA membranes modified with PO_2_. These membranes showed good potential for the regeneration of bone defects when applied to skull defects in an animal model.
(2) Compared to the untreated PLGA barriers, PLGA/PO_2_/HA membranes promote higher osteosynthetic activity, new bone formation and mineralisation levels that are comparable to those of the original bone tissue.
(3) Further investigations of the new membranes in humans are required to develop new techniques that might improve the aesthetic and functional features of future restorations.

## Figures and Tables

**Table 1:** Atomic chemical composition percentages of the HA surface.

Atomic	C1s	O1s	Ca2p	P2p
**Composition (%)**	23.6	44.8	18.1	13.5

**Table 2:** Studied variables in vitro cell cultures.

	PLGA (Control)	PLGA/PO_2_/HA (Experimental)	Statistical Significance
Cell area (μm^2^)	288 ± 124	379 ± 110	
DAPI (nuclei area) (μm^2^)	2.82 ± 2.0	2.55 ± 1.9	
Probe JC-1 (red/green ratio)	1.69 ± 0.41	2.57 ± 0.09	*p* = 0.06
Cells Viability (%)	62.4 ± 6.2	78.2 ± 3.4	*p* < 0.05
Total cells (cells)	2.1 × 10^5^ ± 0.3	4.6 × 10^5^ ± 0.4	*p* < 0.05

**Table 3:** Bone J analysis plugins. (* *p* ≤ 0.05).

	PLGA (Control)	PLGA/PO_2_/HA (Experimental)	Statistical Significance
Bone density (HU)	969.51 ± 145.7	1036.71 ± 241.3	
Bone density (%)	0.59 ± 0.08	0.63 ± 0.14	
Bone Surface (pixels ^2^)	5079.09 ± 1779.49	11,049.51 ± 4304.57	*
Mean trabecular thickness (pixels)	5.74 ± 1.24	6.29 ± 1.52	
Max. trabecular thickness (pixels)	9.13 ± 1.60	11.30 ± 1.75	*
Bone volume (pixels ^2^)	25,016.20 ± 9922.46	45,526.20 ± 15,275.48	*
Total volume (pixels ^2^)	8,120,601.20 ± 16,432.30	8,090,300.45 ± 17,742.30	*
Bone volume/Total volume	0.003 ± 0.001	0.005 ± 0.001	*
Euler characteristic	−34.05 ± 17.49	−74.55 ± 36.65	*
Maximum branch length (pixels)	38.89 ± 7.55	35.47 ± 12.38	
Connectivity (mm^−3^)	35.25 ± 17.45	75.75 ± 36.63	*
Number of branches (branches)	152.45 ± 83.62	265.70 ± 109.02	*
Number of junctions (junctions)	77.45 ± 43.02	139.70 ± 58.92	*
Number of end-point voxels (voxels)	41.20 ± 23.63	51.70 ± 17.78	0.08
Number of junctions voxels (voxels)	180.45 ± 97.38	323.70 ± 132.25	*
Number of slab voxels (voxels)	761.95 ± 434.86	1269.95 ± 467.48	*
Average branch length (pixels)	8.87 ± 1.67	8.64 ± 1.62	
Number of triple points (points)	97.20 ± 40.15	53.450 ± 30.40	*
Number of quadruple points (points)	17.45 ± 10.96	31.95 ± 14.74	*

**Table 4:** Histomorphometric study (* *p* ≤ 0.05).

	PLGA (Control)	PLGA/PO_2_/HA (Experimental)	Statistical Significance
Bone height (μm)	1718 ± 775	1729 ± 700	
Trabecular area (μm^2^)	129,558.45 ± 619,632.90	160,339.46 ± 654,020.23	
Trabecular perimeter (μm)	1282.57 ± 2525.41	1491.46 ± 2747.67	
Number of trabeculae (trabeculae)	27.75 ± 18.81	33.50 ± 33.40	
Fluorescence (μm^2^)	12.43 ± 6.61	14.11 ± 4.82	
Median trabecular area (μm^2^)	129,139.75 ± 618,508.04	160,339.47 ± 654,020.24	
Total trabecular area (μm^2^)	235,428.77 ± 638,030.19	186,611.42 ± 398,159.18	
Osteoid area (μm^2^)	4369.21 ± 8129.87	5974.17 ± 10,159.57	
% Osteoid area/ Total area (%)	0.0501 ± 0.0675	0.0854 ± 0.1172	*
% Bone area von Kossa / Total area (%)	18.72 ± 12.27	17.63 ± 10.60	
Mean trabecular width (μm)	98.19 ± 172.98	106.70 ± 191.84	
% Bone area von Kossa / Total area (%)	18.72 ± 12.27	17.63 ± 10.60	
Mean trabecular width (μm)	98.19 ± 172.98	106.70 ± 191.84	
